# Biomedical and health informatics teaching in Portugal: Current status

**DOI:** 10.1016/j.heliyon.2023.e14163

**Published:** 2023-03-14

**Authors:** Paulo Dias Costa, João Almeida, Sabrina Magalhães Araujo, Patrícia Alves, Ricardo Cruz-Correia, Kaija Saranto, John Mantas

**Affiliations:** a Faculty of Medicine, University of Porto, Porto, Portugal; b Center for Health Technology and Services Research, Porto, Portugal; c Department of Community Medicine, Information and Decision Sciences, Faculty of Medicine, University of Porto, Porto, Portugal; d eMAIS: Movimento Associação dos Sistemas de Informação em Saúde, Portugal; e Faculty of Psychology and Education Sciences, University of Porto, Porto, Portugal; f Center for Research and Intervention in Education, University of Porto, Porto, Portugal; g University of Eastern Finland, Kuopio, Finland; h National and Kapodistrian University of Athens, Athens, Greece

**Keywords:** Education, Teaching, Professional education, Informatics, Medical informatics, Biomedical and health informatics

## Abstract

**Background:**

The domain of Biomedical and Health Informatics (BMHI) lies in the intersection of multiple disciplines, making it difficult to define and, consequently, characterise the workforce, training needs and requirements in this domain. Nevertheless, to the best of our knowledge, there isn’t any aggregated information about the higher education programmes in BMHI currently being delivered in Portugal, and which knowledge, skills, and competencies these programmes aim to develop.

**Aim:**

Our aim is to map BMHI teaching in Portugal. More specifically, our objective is to identify and characterise the: a.) programmes delivering relevant BMHI teaching; b.) geographical distribution and chronological evolution of such programmes; and c.) credit distribution and weight.

**Methods:**

We conducted a descriptive, cross-sectional study to systematically identify all programmes currently delivering any core BMHI modules in Portugal. Our population included all graduate-level programmes being delivered in the 2021/2022 academic year in any Portuguese higher education institution.

**Results:**

We identified 23 programmes delivering relevant teaching in BMHI in Portugal. Of these, eight (35%) were classified as dedicated educational programmes in BMHI, mostly delivered in polytechnic institutes at a master’s level (5; 63%) and located preferentially in the northern part of the country (7). Currently, there are four programmes with potential for accreditation but still requiring some workload increase in certain areas in order to be eligible.

## 1 Introduction

Finding a universal definition of informatics applied to the biomedical and healthcare domain is a complex and challenging task, not only because of the distinct fields it entails [[1], [2], [3]] but also because of the multitude of existing definitions of the sub-disciplines within the domain [[4], [5], [6]], scientific contents, and goals [7]. The domain of Biomedical and Health Informatics (BMHI) lies in the intersection of clinical sciences, public health, computer science, information & communication sciences, chemoinformatics, nanoinformatics, bioinformatics & computational biology, and biomedical engineering [2,8]. This range of diverse fields reflects the nature of BMHI and the apparent confusion underlying the various definitions but, more importantly, it reflects an overlap and convergence of approaches [5,9,10], suggesting that we should view this domain as a “unified discipline with multiple areas of application” [11].

However, this multitude of definitions, sub-disciplines, and scope makes it difficult to characterise the workforce [12] and heavily impacts the definition of training needs and requirements in this domain [8]. In the United States several associations have already addressed training standards [6,13,14], driven by the federal government’s recognition of this domain’s ability to improve the population’s health, mitigate disease complications, and reduce healthcare costs [[15], [16], [17]]. Europe has followed a similar path, with some research being conducted around the training needs and requirements of medical and biomedical informatics students [18,19]. In Portugal, the first two undergraduate ‘Bioinformatics’ programs were introduced in 2003/2004, albeit with focus on Biochemistry, followed in 2005/2006 by the first three ‘Health Informatics’ programmes [20]. By 2009 three other postgraduate programs were introduced, two of which were discontinued in the meantime [21].

Whereas the digital transformation in healthcare is re-shaping professional practices [22], the inclusion of BMHI learning outcomes in education and continuous training of a wide range of healthcare professionals (health informaticians, clinical, health record administrators, healthcare managers, computer scientists/informaticians or other professionals working in the healthcare sector) has been considered paramount [8]. In this context, there have been efforts to define specific sets of BMHI knowledge and competencies required for healthcare professionals [8,[23], [24], [25]], which may support the decisions of students and professionals regarding their education and training, improve employers' awareness about graduates' competencies, and underpin higher education institutions decision making regarding the design, assessment, and improvement of the curriculum of educational programs in the field [26]. Nevertheless, even if studies mapping the integration of BMHI related learning outcomes in the education and training of clinical practitioners exist [25,27], to the best of our knowledge there is no aggregated information about the higher education programmes in BMHI currently being delivered in Portugal, and which knowledge and competencies these programmes aim to develop. By contributing to a better understanding of the educational landscape and training needs in this specific area, which is one of the starting points of curriculum design, filling this knowledge gap may enable better choices for students, employers, and policymakers [28].

As such, our aim is to map BMHI teaching in Portugal. More specifically, our objective is to identify and characterise the: a.) programmes delivering relevant BMHI teaching; b.) geographical distribution and chronological evolution of such programmes; and c.) credit distribution and weight.

## 2 Methods

### 2.1 Study design

We conducted a descriptive cross-sectional study to systematically identify all programmes currently delivering any core BMHI modules in Portugal. Our population included all graduate-level programmes being delivered in the 2021/2022 academic year in any Portuguese higher education institution.

### 2.2 Data collection

The main variables collected were a.) programme general data (including area of education and training area & code; awarding institution’s name, code & location; and co-teaching agreement); b.) programme specific data (such as name; education level & education system/subsystem; and start date); and c.) teaching details (programme duration and credits; official website link to programme structure and syllabus; and module structure, syllabus & credits).

The variables were extracted from the Portuguese Directorate General of Education and Science Statistics (‘Direção-Geral de Estatísticas da Educação e Ciência’- DGEEC^1^) database^2^ on the 12th April 2022, using the National Classification of Education and Training Areas (‘Classificação Nacional das Áreas de Educação e Formação’- CNAEF^3^) which is based in the United Nations Educational, Scientific and Cultural Organization (UNESCO) International Standard Classification of Education (ISCED).^4^ Additionally, the name of the institution was mapped against the code of the institution/school using the Portuguese Directorate General for Higher Education (‘Direção-Geral do Ensino Superior’- DGES^5^) codes,^6^ to obtain all institutions/schools identification codes, location, and contact details.

Module details (such as title, type, structure, syllabus, contents, and credits) were collected by manually analysing the official programme’s webpage during April and May 2022.

The awarding institution and/or the Portuguese DGES were contacted directly with regards to programmes in which data was missing, to confirm eligibility and/or obtain additional details.

### 2.3 Data classification

To systematically retrieve information, we used a three-step methodological approach, as follows.

*Step 1*

We started by identifying all higher education programmes currently being delivered in Portugal in which the core teaching areas were life sciences or related areas, informatics or related areas, or a combination of both. To achieve this, we searched the DGEEC to find all relevant programmes based on the CNAEF codes and areas of education & training description. All programmes included in the codes/areas listed in Table 1 were included. All programmes that did not met the inclusion criteria or had any of the following criteria were excluded from the study: programmes with award recognition pending; further education training courses; technological specialization courses; higher education courses not conferring a degree; professional technical training courses; higher education specialised studies diploma; and preparatory cycle courses.

*Step 2*

**Table 1:** CNAEF codes and areas of education and training used to query the DGEEC.

**AREAS OF EDUCATION & TRAINING**
**CODE - AREA OF EDUCATION & TRAINING** 420 - Life Sciences 421 - Biology and Biochemistry 429 - Life Sciences: not classified elsewhere 440 - Physics Sciences 441 - Physics 449 - Physics Sciences: not classified elsewhere 460 - Mathematics and Statistics 461 - Mathematics 462 - Statistics 469 - Mathematics and Statistics: not classified elsewhere 480 - Informatics 481 - Informatics Sciences 482 - End-User Informatics 489 - Informatics: not classified elsewhere 520 - Engineering and related techniques 524 - Chemical Processes Technology 529 - Engineering and related techniques: not classified elsewhere 720 - Health 721 - Medicine 723 - Nursing 724 - Dental Sciences 725 - Healthcare Sciences 726 - Therapy and Rehabilitation 727 - Pharmacy/Pharmaceutical Sciences 729 - Health: not classified elsewhere 853 - Public Health Services

**Caption: CNAEF** - Classificação Nacional das Áreas de Educação e Formação (*National Classification of Education and Training Areas*); **DGEEC** - Direcção-Geral de Estatísticas da Educação e Ciência (*Directorate General of Education and Science Statistics*).

Next we identified all programmes that matched relevant BMHI areas by combining and expanding different terms relevant to BMHI, based on the related fields recommended by the International Medical Informatics Association (IMIA)[8] - Table 2.

**Table 2:** Main fields of study descriptors used to identify relevant BMHI-related programmes.

**IMIA MAIN FIELDS**	**EXPANDED FIELDS AND TERMS**
Bioinformatics & computational biology Biomedical engineering Chemoinformatics Clinical sciences Computer science Information & communication sciences Nanoinformatics Public health	Medical Information Biomedicine Molecular Biology Life Science Informatics/Technology Biomedical Informatics/Technology Clinical Informatics/Technology Health Informatics/Technology/Engineering Medical Informatics/Technology/Engineering/Electronics Nursing Informatics Big Data in Healthcare Clinical Informatics/Technology/Engineering Clinical Data Management Data Mining in Healthcare/Medicine Internet of Things in Healthcare/Medicine Wireless Networks in Healthcare/Medicine Analytics in Healthcare/Medicine Digital Health Systems E-Health Telemedicine Imaging

**Caption: BMHI** - Biomedical and Health Informatics; **IMIA** - International Medical Informatics Association.

Given the many different terms used in this domain, we have built a broad-spectrum query by using permutations and wildcards, in Portuguese, of the keywords ‘health’, ‘clinical’, ‘medical’, ‘medicine’, ‘nursing’, ‘computational’, ‘bioinformatics', ‘bioengineering’, ‘biomedical’, ‘informatics’, ‘data’ and ‘science’; the query used is detailed in Appendix I.

Programme titles that matched any of the relevant areas previously defined in Table 2 were included.

*Step 3*

In this step, the programme’s structure and syllabus was assessed to identify relevant BMHI topics being delivered, and all selected programmes classified into a main area.

To achieve this, three unblinded reviewers (PDC, JA and SMA) with formal BMHI training and experience accessed each programme’s official website, and independently screened all module’s syllabus for alignment with the domains defined by the IMIA (Table 3 [8]). Each module was then assigned a domain and the corresponding European Credit Transfer System (ECTS)^7^ credits. Finally, each programme was allocated to a main area of education corresponding to the domain with most credits. Any discrepancies between the reviewers were resolved through discussion, by jointly analysing the course structure and syllabus.

**Table 3:** Domains used to classify individual modules within relevant BMHI programmes.

**DOMAIN**
Biomedical and Health Informatics Core Knowledge and Skills Medicine, Health and Biosciences, Health System Organization Informatics/Computer Science, Mathematics, Biometry Optional Modules in BHMI and from Related Fields

Adapted from [8].

**Fig. 1:**
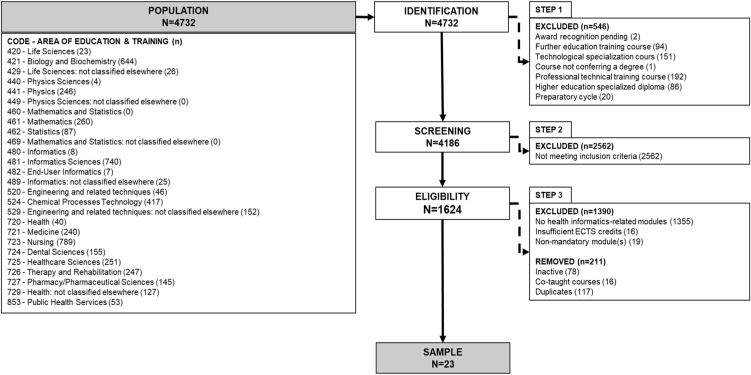
Selection process flowchart.

Programmes that delivered at least one mandatory core BMHI module with a minimum of four ECTS were included, following the IMIA recommendation on minimum student workload [8]. All programs that did not meet the inclusion criteria or that were inactive in the current academic year were excluded from the study. To ensure data quality, consistency, and avoid over-representation of programmes, duplicate courses were also removed (e.g. postgraduate diplomas that were part of a master’s programme, or when multiple pathways to achieve the same degree existed such as integrated master’s). Similarly, co-taught programmes, delivered in multiple schools of the same institution or involving multiple institutions, were removed and only one entry was kept. Fig. 1 below illustrates the full selection process.

### 2.4 Data analysis

Data was extracted in .xls format using Microsoft Excel (Microsoft Corporation, Redmond, Washington, USA), and compiled and organised in a single dataset using RStudio IDE (RStudio, Boston, Massachusetts, USA). Statistical analysis, tables, and graphics were performed using R (R Foundation for Statistical Computing, Vienna, Austria) on RStudio IDE (RStudio, Boston, Massachusetts, USA).

Categorical variables are presented as absolute and relative frequencies. Quantitative variables are presented as mean and standard-deviation or as median and interquartile range, depending on the distribution.

## 3 Results & discussion

### 3.1 General characterisation

We identified 23 programmes delivering relevant teaching in BMHI in Portugal. A characterisation of all programmes is summarised in Table 6.

Regarding main area of education, nine programmes (39.1%) were classified as having BMHI modules as part of ‘Medical, Nursing, Health Care Management, Dentistry, Pharmacy and Public Health’ (MNHDPP) programmes, whereas eight programmes (34.8%) were classified as dedicated educational programmes in BMHI. The remaining six (26.1%) were classified as having BMHI modules as part of ‘Informatics, Computer Science Programs and Engineering’ (ICSE) programmes - Table 4.

**Table 4:** Programme’s main area of education.

**TOTAL (N** = **23)**	
BMHI ICSE MNHDPP	8 (34.8%) 6 (26.1%) 9 (39.1%)

**Caption: BMHI** - Biomedical and Health Informatics; **ICSE** - Informatics, Computer Science Programs and Engineering; **MNHDPP** - Medical, Nursing, Health Care Management, Dentistry, Pharmacy and Public Health.

When comparing CNAEF codes with the main areas resulting from the domains credits addition it was possible to identify some heterogeneity between the classifications. For example, programmes classified in CNAEF areas so distinct as ‘Chemical Processes Technology’ and ‘Biology and Biochemistry’ were classified in the ICSE main area. In contrast, other programmes in the ‘Biology and Biochemistry’ CNAEF area were classified in the MNHDPP main area. The updated CNAEF classification (which is harmonised with EUROSTAT^8^ and CEDEFOP^9^) can include a primary and two secondary categories to classify programmes; however only the primary code is currently used. As a consequence, BMHI programmes are classified within a variety of codes and areas (e.g. biology and biochemistry, chemical processes technology, or healthcare sciences) that might not reflect the true nature of BMHI. As such, it would be interesting to update CNAEF classifications and reach a European consensus, possibly involving the European Federation of Medical Informatics (EFMI), to define/create a standardised classification for all BMHI-related programmes. Table 5 below provides additional information on this topic.

**Table 5:** Programme’s CNAEF code/area and main area classification comparison.

	BMHI (N = 8)	ICSE (N = 6)	MNHDPP (N = 9)	TOTAL (N = 23)
CNAEF Code - Area				
462 - Statistics	1 (12.5%)	0 (0%)	0 (0%)	1 (4.4%)
481 - Informatics Sciences	1 (12.5%)	0 (0%)	0 (0%)	1 (4.4%)
489 - Informatics: not classified	1 (12.5%)	0 (0%)	0 (0%)	1 (4.4%)
524 - Chemical Processes Technology	1 (12.5%)	1 (16.7%)	0 (0%)	2 (8.7%)
720 - Health	2 (25.0%)	0 (0%)	1 (11.1%)	3 (13.0%)
725 - Healthcare Sciences	1 (12.5%)	0 (0%)	3 (33.3%)	4 (17.4%)
729 - Health: not classified elsewhere	1 (12.5%)	0 (0%)	0 (0%)	1 (4.4%)
420 - Life Sciences	0 (0%)	2 (33.3%)	0 (0%)	2 (8.7%)
421 - Biology and Biochemistry	0 (0%)	1 (16.7%)	2 (22.2%)	3 (13.0%)
520 - Engineering and related techniques	0 (0%)	1 (16.7%)	0 (0%)	1 (4.4%)
529 - Engineering and related techniques: not classified elsewhere	0 (0%)	1 (16.7%)	0 (0%)	1 (4.4%)
721 - Medicine	0 (0%)	0 (0%)	3 (33.3%)	3 (13.0%)

**Caption: BMHI** - Biomedical and Health Informatics; **CNAEF** - Classificação Nacional das Áreas de Educação e Formação (*National Classification of Education and Training Areas*); **ICSE** - Informatics, Computer Science Programs and Engineering; **MNHDPP** - Medical, Nursing, Health Care Management, Dentistry, Pharmacy and Public Health.

**Table 6:** Summary of the general characteristics of programmes delivering relevant BMHI teaching.

Main Area	Programme Name	Institution	Location	Education System	Education Subsystem	Level	Start Date	Duration	ECTS Credits	Co-taught
BMHI	Applied Health Sciences - Community Intervention and Biotechnology (biotechnology branch)	Instituto Politécnico de Bragança	Bragança	Public	Polytechnic	MSc	2019	3	90	No
BMHI	Biostatistics and Bioinformatics Applied to Healthcare	Instituto Politécnico do Porto	Porto	Public	Polytechnic	MSc	2016	4	120	No
BMHI	Biomedical Engineering (medical informatics branch)	Universidade do Minho	Braga	Public	University	MSc	2021	4	120	No
BMHI	Medical Technology and Healthcare Business	Instituto Politécnico do Porto	Porto	Public	Polytechnic	MSc	2017	4	120	No
BMHI	Health Data Science	Instituto Politécnico de Lisboa	Lisbon	Public	Polytechnic	PGC	2021	2	60	No
BMHI	Nursing Information Systems	Escola Superior de Enfermagem do Porto	Porto	Public	Polytechnic	PGC	2009	2	30	No
BMHI	Health Data Science (health informatics branch)	Universidade do Porto	Porto	Public	University	PhD	2018	8	240	No
BMHI	Medical Informatics	Universidade do Porto	Porto	Public	University	MSc	2011	4	120	Yes
ICSE	Bioinformatics	Instituto Politécnico de Setúbal	Setúbal	Public	Polytechnic	BSc	2016	6	180	Yes
ICSE	Bioengineering in Regenerative and Precision Medicine (precision medicine branch)	Universidade de Lisboa	Lisbon	Public	University	MSc	2021	4	120	No
ICSE	Bioinformatics and Applications to Life Sciences (branch applied computing)	Universidade de Trás-os-Montes e Alto Douro	Vila Real	Public	University	MSc	2017	4	120	No
ICSE	Clinical Bioinformatics (clinical decision support branch)	Universidade de Aveiro	Aveiro	Public	University	MSc	2020	4	120	No
ICSE	Medical Informatics Engineering	Instituto Politécnico do Cávado e do Ave	Barcelos	Public	Polytechnic	BSc	2018	6	180	No
ICSE	Biomedical Engineering and Biophysics	Universidade de Lisboa	Lisbon	Public	University	MSc	2020	4	120	No
MNHDPP	Biomedical Sciences	Instituto Universitário de Ciências da Saúde	Paredes	Private	University	BSc	2010	6	180	No
MNHDPP	Bioinformatics (information technologies branch)	Universidade do Minho	Braga	Public	University	MSc	2011	4	120	No
MNHDPP	Medical Imaging and Radiotherapy	Instituto Politécnico da Lusofonia	Lisbon	Private	Polytechnic	BSc Hons	2018	8	240	No
MNHDPP	Applied Biomedicine (computation branch)	Universidade Católica Portuguesa	Viseu	Private	University	MSc	2021	4	120	No
MNHDPP	Healthcare Policies, Management and Evaluation	Universidade do Porto	Porto	Public	University	PGC	2020	2	60	No
MNHDPP	Medical Imaging and Radiotherapy	Instituto Politécnico de Coimbra	Coimbra	Public	Polytechnic	BSc Hons	2014	8	240	No
MNHDPP	Medical Imaging and Radiotherapy	Universidade do Algarve	Faro	Public	Polytechnic	BSc Hons	2014	8	240	No
MNHDPP	Medicine	Universidade do Porto	Porto	Public	University	iMSc	2011	12	360	No
MNHDPP	Medicine (analytical data systems branch)	Universidade do Minho	Braga	Public	University	iMSc	2011	12	360	No

**Caption: BMHI** - Biomedical and Health Informatics; **BSc** - Bachelor Bachelor of Science; BSc Hons - Bachelor Bachelor of Science with Honours; **ICSE** - Informatics, Computer Science Programs and Engineering; **iMSc** - Integrated Master of Science; **MNHDPP** - Medical, Nursing, Health Care Management, Dentistry, Pharmacy and Public Health; **MSc** - Master of Science; **PhD** - Philosophy Doctor; **PGC** - Postgraduate Certificate.

Overall, most of the programmes identified were taught in public higher education institutions (20; 87.0%), mostly within universities (13; 56.5%), at a master’s level (13; 56.5%). When considering programmes by main area, these trends are very similar except for dedicated BHMI programmes being delivered mostly in polytechnic institutes (5; 62.5%). Portuguese higher education is a binary system, in which universities are mainly focused on providing a solid theoretical background and oriented to promote research and produce knowledge, whereas polytechnic institutes are mainly oriented towards applied research and aim at providing a solid technical/practical approach to problem-solving [29]. Nevertheless, with the introduction of the Bologna process these distinctions are progressively more blurred, suggesting that the reasons behind certain programmes are being delivered in universities or polytechnic institutes are related to local needs and teaching expertise, as well as to the profile of the recent graduate (i.e. possessing a more theoretical or a more hands-on approach) [30].

The median programme’s ECTS was 120, except for MNHDPP programmes in which the median ECTS credits was 240. These results highlight that MNHDPP programmes have a longer duration (probably underpinned by the longer duration of Medicine and Healthcare Sciences programmes) and are mostly taught at a BSc level. On the other hand, dedicated BMHI programmes are often of shorter duration, and taught at a master’s level. Although there is no evidence to support these findings, it seems to suggest that BMHI workforce needs are being provided by professionals from diverse backgrounds (healthcare and/or informatics) who develop some BHMI competencies in their graduate degrees but to work in BHMI they may need further specialization.

A summary of these results is presented in Table 7.

**Table 7:** Programme’s education system, sub-system, level and ECTS credits.

	BMHI (N = 8)	ICSE (N = 6)	MNHDPP (N = 9)	TOTAL (N = 23)
**Education System**				
Public	8 (100%)	6 (100%)	6 (66.7%)	20 (87.0%)
Private	0 (0%)	0 (0%)	3 (33.3%)	3 (13.0%)
**Education Subsystem**				
Polytechnic	5 (62.5%)	2 (33.3%)	3 (33.3%)	10 (43.5%)
University	3 (37.5%)	4 (66.7%)	6 (66.7%)	13 (56.5%)
**Level**				
MSc	5 (62.5%)	4 (66.7%)	2 (22.2%)	11 (47.8%)
PGC	2 (25.0%)	0 (0%)	1 (11.1%)	3 (13.0%)
PhD	1 (12.5%)	0 (0%)	0 (0%)	1 (4.4%)
BSc	0 (0%)	2 (33.3%)	1 (11.1%)	3 (13.0%)
BSc Hons	0 (0%)	0 (0%)	3 (33.3%)	3 (13.0%)
iMSc	0 (0%)	0 (0%)	2 (22.2%)	2 (8.7%)
ECTS Credits				
Mean (SD)	113 (61.6)	140 (31.0)	213 (104)	159 (86.5)
Median [Min, Max]	120 [30; 240]	120 [120; 180]	240 [60; 360]	120 [30; 360]

**Caption: BMHI** - Biomedical and Health Informatics; **BSc** - Bachelor Bachelor of Science; **BSc Hons** - Bachelor Bachelor of Science with Honours; **ECTS** - European Credit Transfer System; **ICSE** - Informatics, Computer Science Programs and Engineering; **iMSc** - Integrated Master of Science; **MNHDPP** - Medical, Nursing, Health Care Management, Dentistry, Pharmacy and Public Health; **MSc** - Master of Science; **PhD** - Philosophy Doctor; **PGC** - Postgraduate Certificate; **SD** - Standard Deviation.

### 3.2 Geographical distribution

**Fig. 2:**
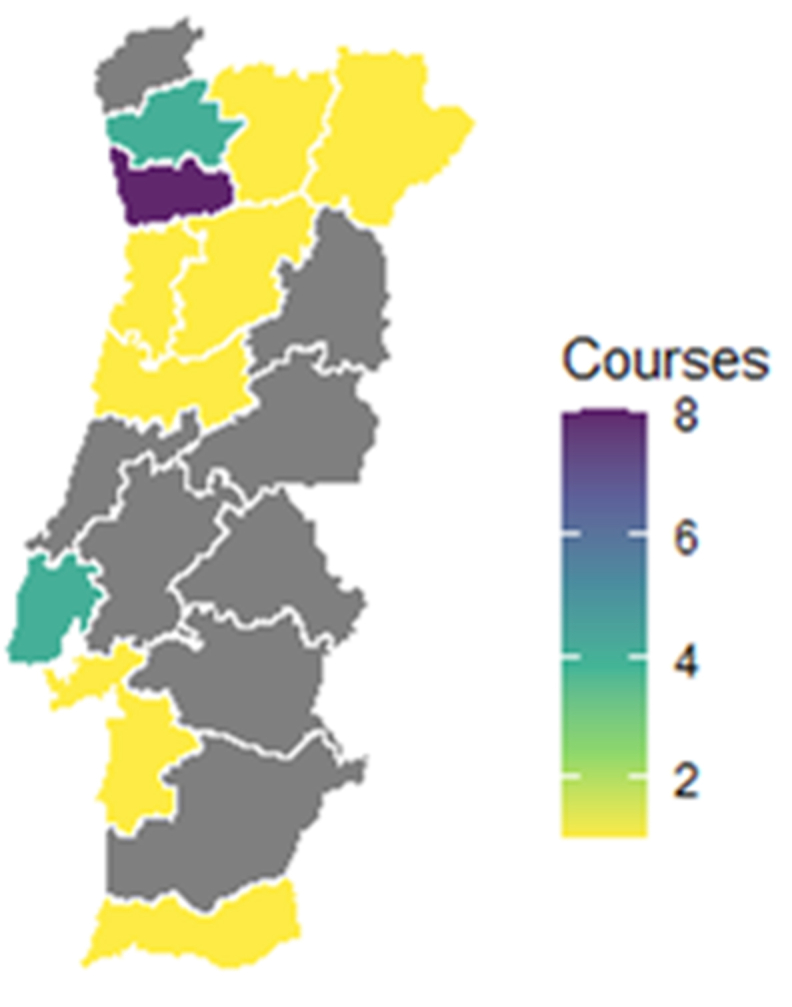
Geographical distribution, by district, of all programmes.

When looking at the distribution by district, it is visible a higher concentration of programmes in the northern part of the country (Fig. 2).

**Fig. 3:**
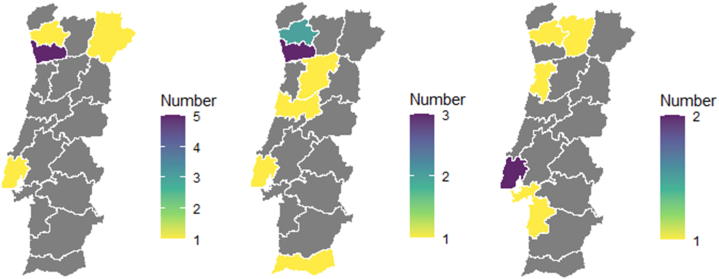
Geographical distribution, by district, of programmes in the areas of Biomedical and Health Informatics (A), Medical, Nursing, Health Care Management, Dentistry, Pharmacy and Public Health (B) and Informatics, Computer Science and Engineering (C).

Dedicated BMHI programmes are mostly located in the northern part of the country (Fig. 3A). MNHDPP (Fig. 3B) and ISCE (Fig. 3C) programmes, on the other hand, have a more widespread distribution, spreading from the northern part of the country to the southmost part of the country, but mainly along the coast.

This distribution is in line with the data provided by the DGES [31], which indicates that most institutions and programmes are located along the coast, mostly around major cities. However, the reasons behind the geographical distribution of BMHI programmes, mostly located in the northern part of the country, are not clear but may suggest that there is a higher concentration of specialist teaching or research clusters in that region and that although students are trained here, they may join the workforce elsewhere. Furthermore, the narrowing of educational regional disparities in the area of BHMI through the strengthening of the training offer, namely in peripheral regions, could improve educational opportunities for the population of these regions (e.g. potential students who do not wish/want to engage in regional mobility), and contribute to the strengthening of human capital, potentially contributing to the socio-economic development of these regions [32].

### 3.3 Temporal distribution

**Fig. 4:**
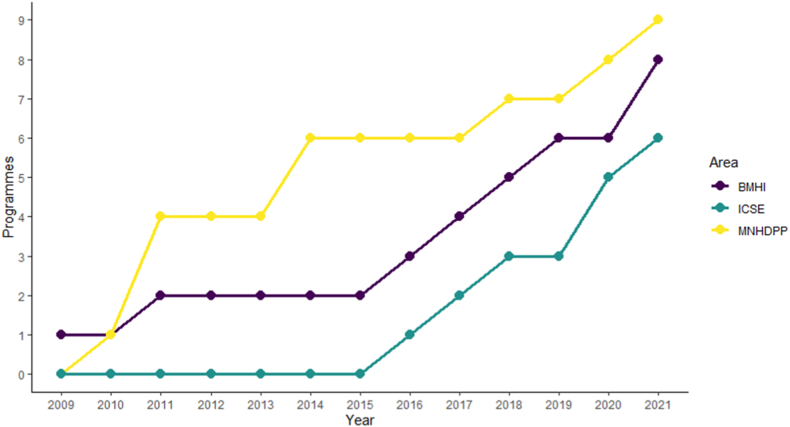
Cumulative number of Biomedical and Health Informatics (purple line), Medical, Nursing, Health Care Management, Dentistry, Pharmacy and Public Health (yellow line) and Informatics, Computer Science and Engineering (green line) programmes by year.

There seems to be a prominent emergence of MNHDPP programmes delivering BHMI-related teaching from 2009 to 2014, accompanied, in that same period, by a low emergence of BMHI programmes and a stagnant emergence of ICSE programmes delivering BHMI-related teaching. By 2015, however, that scenario was reversed, with the rapid emergence of several BMHI programmes and ICSE programmes delivering BHMI-related teaching. Since 2019 there seems to be an emergence of new programmes in all areas, reaching an all-time high number of programmes delivering core BMHI teaching in 2021. At this point it is also important to emphasise that in the last 12 years we have seen the emergence of 22 new programmes delivering core BMHI teaching. Fig. 4 illustrates these findings.

These findings are consistent with the growth of the Portuguese higher education system potentiated by the Bologna process between 2006 and 2012; similarly, the stringent economic measures imposed between 2013 and 2015 could explain the deceleration of programmes growth [33]. Finally, a possible explanation for the growth of BMHI programmes since 2015 might be the implementation of the National Health Plan 2011–2016 regarding information and communication technologies [34], and again in the last two years, supported by the European Commission’s Recovery and Resilience Facility for digital transition [35], perhaps reflecting the growing demand for professionals with these skills.

### 3.4 Credits distribution

With regards to dedicated BMHI programmes, the programmes delivering the most substantial core BMHI teaching are the ‘Medical Informatics’ programme at Universidade do Porto and the ‘Biomedical Engineering (medical informatics branch)’ at Universidade do Minho, while providing relevant teaching in informatics but with reduced workload in the medical domain. These two programmes can, potentially, be eligible for accreditation with the IMIA. To fully match the criteria, however, the former needs to increase the workload on the medical domain while the later needs to increase the workload on both the core BMHI and medical domains.

The ‘Medical Informatics Engineering’ programme at Instituto Politécnico do Cávado e do Ave delivers relevant and balanced teaching in all relevant domains in the ICSE programmes with BMHI modules category. Nevertheless, to fully match the criteria of IMIA, a workload increase on core BMHI teaching is needed.

Similarly, for BMHI modules as part of MNHDPP programmes, the ‘Medicine (analytical data systems branch)’ at Universidade do Minho programme provides robust teaching in all relevant domains, with strong emphasis in the medical domain, and is at present the only programme that meets current criteria for IMIA accreditation in its main area.

A summary of mandatory and optional ECTS credits for all programmes delivering relevant BMHI teaching is presented in Table 8.

**Table 8:** Summary of mandatory and optional ECTS credits, by domain, attributed to programmes delivering relevant BMHI teaching.

			MANDATORY ECTS					OPTIONAL ECTS				
Main Area	Programme Name	Institution	BMHI	Medical	Informatics	Sum	Total	BMHI	Medical	Informatics	Sum	Total
BMHI	Applied Health Sciences - Community Intervention and Biotechnology (biotechnology	Instituto Politécnico de Bragança	7	0	3,5	10,5	90	0	0	0	0	0
BMHI	Biostatistics and Bioinformatics Applied to Healthcare	Instituto Politécnico do Porto	15	0	7,5	22,5	105	0	7,5	7,5	15	15
BMHI	Biomedical Engineering (medical informatics branch)	Universidade do Minho	60	0	20	80	120	0	0	0	0	0
BMHI	Medical Technology and Healthcare Business	Instituto Politécnico do Porto	15	10	5	30	120	0	0	0	0	0
BMHI	Health Data Science	Instituto Politécnico de Lisboa	21	4	21	46	60	0	0	0	0	0
BMHI	Nursing Information Systems	Escola Superior de Enfermagem do Porto	28	0	0	28	28	6	0	0	6	2
BMHI	Health Data Science (health informatics branch)	Universidade do Porto	33	9	6	48	240	0	0	0	0	0
BMHI	Medical Informatics	Universidade do Porto	81	6	21	108	108	6	0	6	12	12
ICSE	Bioinformatics	Instituto Politécnico de Setúbal	5	10	66,5	81,5	180	0	0	0	0	0
ICSE	Bioengineering in Regenerative and Precision Medicine (precision medicine branch)	Universidade de Lisboa	6	6	6	18	120	0	0	0	0	0
ICSE	Bioinformatics and Applications to Life Sciences (branch applied computing)	Universidade de Trás-os-Montes e Alto Douro	6	6	24	36	120	0	0	0	0	0
ICSE	Clinical Bioinformatics (clinical decision support branch)	Universidade de Aveiro	6	12	24	42	114	0	18	36	54	6
ICSE	Medical Informatics Engineering	Instituto Politécnico do Cávado e do Ave	33	15	78	126	180	0	0	0	0	0
ICSE	Biomedical Engineering and Biophysics	Universidade de Lisboa	6	0	12	18	78	24	6	6	36	42
MNHDPP	Biomedical Sciences	Instituto Universitário de Ciências da Saúde	4	56	8	68	180	0	0	0	0	0
MNHDPP	Bioinformatics (information technologies branch)	Universidade do Minho	5	10	5	20	120	0	0	0	0	0
MNHDPP	Medical Imaging and Radiotherapy	Instituto Politécnico da Lusofonia	5	28	5	38	240	0	0	0	0	0
MNHDPP	Applied Biomedicine (computation branch)	Universidade Católica Portuguesa	6	60	17	83	120	0	0	0	0	0
MNHDPP	Healthcare Policies, Management and Evaluation	Universidade do Porto	6	18	0	24	42	9	27	6	42	18
MNHDPP	Medical Imaging and Radiotherapy	Instituto Politécnico de Coimbra	4	10	3	17	240	0	0	0	0	0
MNHDPP	Medical Imaging and Radiotherapy	Universidade do Algarve	4	18	3	25	240	0	0	0	0	0
MNHDPP	Medicine	Universidade do Porto	12	330	4	346	346	10	218	4	232	14
MNHDPP	Medicine (analytical data systems branch)	Universidade do Minho	45	300	15	360	360	0	0	0	0	0

**Caption: BMHI** - Biomedical and Health Informatics; **BSc** - Bachelor Bachelor of Science; **BSc Hons** - Bachelor Bachelor of Science with Honours; **ECTS** - European Credit Transfer System; **ICSE** - Informatics, Computer Science Programs and Engineering; **iMSc** - Integrated Master of Science; **MNHDPP** - Medical, Nursing, Health Care Management, Dentistry, Pharmacy and Public Health; **MSc** - Master of Science; **PhD** - Philosophy Doctor; **PGC** - Postgraduate Certificate.

## 4 Conclusions

### 4.1 Limitations

Although we believe this work will be paramount in defining the current scenario of BMHI teaching in Portugal, it is not without limitations.

Firstly, by using a national database that might not be fully updated we might have missed relevant programmes; however, due to time constraints and the predictable difficulty in obtaining a response from higher education institutions should we had used a questionnaire, we decided that this was the most sensible approach. Moreover, we decided to classify individual modules ourselves instead of asking higher education institutions to do it due to the potential for increased inter-observer variability, and hence the risk of bias; by doing so there is, however, the risk of misrepresenting or misclassifying some modules, which we tried to mitigate by joint discussion and strict adherence to the IMIA recommendations [8].

Secondly, we have considered the ‘best case scenario’ (i.e. the scenario that carried the most BMHI-related credits) for branches within a programme and for optional modules within a programme; this assumption offers no guarantee that any relevant BMHI modules will be chosen but taking a different approach would mean that we could be excluding relevant programmes or branches.

Thirdly, and due to the huge number of possibilities, optional modules that allowed students to choose any module from any faculty within the university were not considered.

### 4.2 Future work

Due to the current pandemic state, derived from the Coronavirus disease, we decided not to evaluate teaching delivery modes (such as face-to-face and remote) as there was a risk of over-representing remote teaching/learning and therefore the introduction of bias. In the future we plan to identify teaching delivery modes and assess their impact on the programme’s performance. In the future we also plan to identify the number of IMIA accredited programmes and explore its impact on the programme’s outcomes.

It would also be crucial to understand what presides the decision to start or close some programmes (especially core BMHI), and the reasons behind the growth of BMHI programmes since 2015, perhaps by directly inquiring course directors. Furthermore, it would be important to understand why some programmes are being taught in different education sub-systems, and how that reflects on the educational approach, student’s competencies, and skills.

Since this was a descriptive and exploratory study, it is not clear to us the reason most BMHI programmes were being delivered at master’s level and why they were mainly located in the north region of the country around Porto, and therefore additional research might be warranted.

It would be central to identify the real demands of the market in terms of the number of graduates and understand how graduates' competencies map against the needs of future employers. Establishing employment levels, local industry needs, and destination and salary of recent graduates, in conjunction with the competencies mapping and workforce study proposed earlier, would be paramount to adjust the curriculum, training requirements, and workforce planning.

Finally, and although some work has been done in that respect (although still not available to the public) [36], mapping of education in this area in the European Higher Education Area still remains a gap in current knowledge.

### 4.3 Closing remarks

This is, to the best of our knowledge, the first study to characterise the status of BMHI teaching in Portugal, providing an overview of the number and education level of programmes, the type of education system they are included in, the programme’s geographical distribution and chronological behaviour, and the BMHI-related credit weight. This work allows not only the quantification and characterisation of BMHI programmes in Portugal but, more importantly, might pave the way to a deeper understanding of BMHI scenario in the country.

Although we have found some programmes delivering relevant BMHI teaching, the credit weight of specialist BMHI modules is relatively low; nevertheless, we have identified four programmes with the potential to be accredited, which, considering the size of the country, might be significant. The recently updated guidelines from the IMIA [37] incorporate new knowledge domains (social & behavioral sciences and management science) which may potentially prompt the curriculum revision in some programmes in order to conform to these recommendations.

Furthermore, our study has shown that there is only one PhD programme in the country delivering relevant BMHI teaching, possibly meaning that Portugal might be prepared to build capacity in BMHI for the entry and intermediate levels but the future of the area might be compromised if research in not stimulated, namely by the introduction of new PhD programmes and/or a tighter relationship between academia and industry.

Finally, by openly sharing our final dataset with the community we believe we can facilitate and propel research in this area, while promoting the advancement of BMHI and, ultimately, contribute to better patient care.

## Funding

PDC was supported by the Fundação para a Ciência e a Tecnologia, I.P, through grant number UI/BD/151574/2021. PA was supported by the Fundação para a Ciência e a Tecnologia, I.P, through grant number SFRH/BD/145719/2019. Publication fees were supported by 10.13039/501100006752Universidade do Porto. The funders did not have any role in the data analysis or the article contents. The content of the project is solely the responsibility of the authors and does not necessarily represent the official views of the funders.

## Author statement

All authors conceived and designed the experiments, performed the experiments, analyzed and interpreted the data, and wrote the paper.

## Declaration of competing interest

Paulo Dias Costa, João Almeida, Sabrina Magalhães Araújo, Patrícia Alves and Ricardo Cruz Correia are affiliated with the Faculty of Medicine at University of Porto. João Almeida, Sabrina Magalhães Araújo and Ricardo Cruz Correia are actively involved in teaching delivery to the ‘Medical Informatics’ master’s programme at the University of Porto. Ricardo Cruz Correia is the course director of the ‘Medical Informatics’ master’s programme at the University of Porto. John Mantas is the working group chair on education for the ‘European Federation for Medical Informatics’.

## Footnotes

1 https://www.dgeec.mec.pt.

2 https://cnaef.dgeec.mec.pt/cnaef.php.

3 https://estatistica.dgeec.mec.pt/docs/docs_cdr/%C3%81REAS_EDUCA%C3%87%C3%83O_E_FORMA%C3%87%C3%83O_ISCED_2013_PT_31_03_2017.pdf.

4 https://uis.unesco.org/en/topic/international-standard-classification-education-isced.

5 https://www.dges.gov.pt/en.

6 https://www.dgeec.mec.pt/np4/EstabCursosRamos/%7B$clientServletPath%7D/?newsId=712&fileName=MatrizCesRamos_12_05_2022.xlsx.

7 https://education.ec.europa.eu/education-levels/higher-education/higher-education-initiatives/inclusive-and-connected-higher-education/european-credit-transfer-and-accumulation-system.

8 https://ec.europa.eu/eurostat.

9 https://www.cedefop.europa.eu/en.
