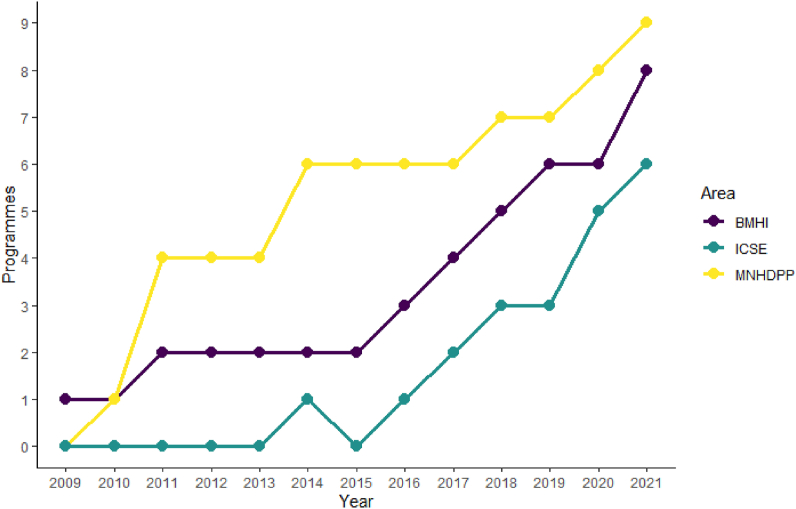# Corrigendum to “Biomedical and health informatics teaching in Portugal: Current status” [Heliyon 9(3) (March 2023) e14163]

**DOI:** 10.1016/j.heliyon.2023.e16208

**Published:** 2023-05-18

**Authors:** Paulo Dias Costa, João Almeida, Sabrina Magalhães Araujo, Patrícia Alves, Ricardo Cruz-Correia, Kaija Saranto, John Mantas

**Affiliations:** a Faculty of Medicine, University of Porto, Porto, Portugal; b Center for Health Technology and Services Research, Porto, Portugal; c Department of Community Medicine, Information and Decision Sciences, Faculty of Medicine, University of Porto, Porto, Portugal; d eMAIS: Movimento Associação dos Sistemas de Informação em Saúde, Portugal; e Faculty of Psychology and Education Sciences, University of Porto, Porto, Portugal; f Center for Research and Intervention in Education, University of Porto, Porto, Portugal; g University of Eastern Finland, Kuopio, Finland; h National and Kapodistrian University of Athens, Athens, Greece

## Changes to the article text

In the Abstract section, Results sub-section, where it reads “We identified 23 programmes delivering relevant teaching in BMHI in Portugal. Of these, eight (35%) were classified […] should read “We identified 24 programmes delivering relevant teaching in BMHI in Portugal. Of these, eight (33%) were classified […]”.

In the ‘Results & Discussion’ section, ‘General Characterisation’ sub-section, where it reads “We identified 23 programmes delivering relevant teaching in BMHI in Portugal. A characterisation of all programmes is summarised in **Table 6**. Regarding main area of education, nine programmes (39%) were classified as having BMHI modules as part of ‘Medical, Nursing, Health Care Management, Dentistry, Pharmacy and Public Health’ (MNHDPP) programmes, whereas eight programmes (34.8%) were classified as dedicated educational programmes in BMHI. The remaining six (26.1%) were classified as having BMHI modules as part of ‘Informatics, Computer Science Programs and Engineering’ (ICSE) programmes - **Table 4**.” should read “We identified 24 programmes delivering relevant teaching in BMHI in Portugal. A characterisation of all programmes is summarised in **Table 6**. Regarding main area of education, nine programmes (37.5%) were classified as having BMHI modules as part of ‘Medical, Nursing, Health Care Management, Dentistry, Pharmacy and Public Health’ (MNHDPP) programmes, whereas eight programmes (33.3%) were classified as dedicated educational programmes in BMHI. The remaining seven (29.2%) were classified as having BMHI modules as part of ‘Informatics, Computer Science Programs and Engineering’ (ICSE) programmes - **Table 4**.”

In the ‘Results & Discussion’ section, ‘General Characterisation’ sub-section, where it reads “Overall, most of the programmes identified were taught in public higher education institutions (20; 87.0%), mostly within universities (13; 56.5%), at a master's level (13; 56.5%).” should read “Overall, most of the programmes identified were taught in public higher education institutions (21; 87.5%), mostly within universities (14; 58.3%), at a master's level (11; 45.8%).”

## Changes to the tables

**Tables 4–8** should be updated as per below. Table titles and captions remain the same.

Table 4

**Table:** 

	TOTAL (N = 24)
BMHI	8 (33.3%)
ICSE	7 (29.2%)
MNHDPP	9 (37.5%)

Table 5

**Table:** 

	BMHI (N = 8)	ICSE (N = 7)	MNHDPP (N = 9)	TOTAL (N = 24)
CNAEF Code - Area				
462 - Statistics	1 (12.5%)	0 (0%)	0 (0%)	1 (4.17%)
481 - Informatics Sciences	1 (12.5%)	0 (0%)	0 (0%)	1 (4.17%)
489 - Informatics: not classified elsewhere	1 (12.5%)	0 (0%)	0 (0%)	1 (4.17%)
524 - Chemical Processes Technology	1 (12.5%)	2 (28.6%)	0 (0%)	3 (12.5%)
720 - Health	2 (25.0%)	0 (0%)	1 (11.1%)	3 (12.5%)
725 - Healthcare Sciences	1 (12.5%)	0 (0%)	3 (33.3%)	4 (16.7%)
729 - Health: not classified elsewhere	0 (0%)	0 (0%)	0 (0%)	1 (4.17%)
420 - Life Sciences	0 (0%)	2 (28.6%)	0 (0%)	2 (8.33%)
421 - Biology and Biochemistry	0 (0%)	1 (14.3%)	2 (22.2%)	3 (12.5%)
520 - Engineering and related techniques	0 (0%)	1 (14.3%)	0 (0%)	1 (4.17%)
529 - Engineering and related techniques: not classified elsewhere	0 (0%)	1 (14.3%)	0 (0%)	1 (4.17%)
721 - Medicine	0 (0%)	0 (0%)	3 (33.3%)	3 (12.5%)

Table 6

**Table:** 

MAIN AREA	PROGRAMME NAME	INSTITUTION	LOCATION	EDUCATION SYSTEM	EDUCATION SUBSYSTEM	LEVEL	START DATE	DURATION (semesters)	ECTS CREDITS	CO-TAUGHT
BMHI	Applied Health Sciences - Com. Intervention and Biotech. (biotechnology branch)	Instituto Politécnico de Bragança	Bragança	Public	Polytechnic	MSc	2019	3	90	No
BMHI	Biomedical Engineering (medical informatics branch)	Universidade do Minho	Braga	Public	University	MSc	2021	4	120	No
BMHI	Biostatistics and Bioinformatics Applied to Healthcare	Instituto Politécnico do Porto	Porto	Public	Polytechnic	MSc	2016	4	120	No
BMHI	Health Data Science	Instituto Politécnico de Lisboa	Lisbon	Public	Polytechnic	PGC	2021	2	60	No
BMHI	Health Data Science (health informatics branch)	Universidade do Porto	Porto	Public	University	PhD	2018	8	240	No
BMHI	Medical Informatics	Universidade do Porto	Porto	Public	University	MSc	2011	4	120	Yes
BMHI	Medical Technology and Healthcare Business	Instituto Politécnico do Porto	Porto	Public	Polytechnic	MSc	2017	4	120	No
BMHI	Nursing Information Systems	Escola Superior de Enfermagem do Porto	Porto	Public	Polytechnic	PGC	2009	2	30	No
ICSE	Bioengineering in Regenerative and Precision Medicine (precision medicine branch)	Universidade de Lisboa	Lisbon	Public	University	MSc	2021	4	120	No
ICSE	Bioinformatics	Instituto Politécnico de Setúbal	Setúbal	Public	Polytechnic	BSc	2016	6	180	Yes
ICSE	Bioinformatics and Applications to Life Sciences (branch applied computing)	Universidade de Trás-os-Montes e Alto Douro	Vila Real	Public	University	MSc	2017	4	120	No
ICSE	Biomedical Engineering	Universidade Nova de Lisboa	Lisbon	Public	University	BSc	2014	6	180	No
ICSE	Biomedical Engineering and Biophysics	Universidade de Lisboa	Lisbon	Public	University	MSc	2020	4	120	No
ICSE	Clinical Bioinformatics (clinical decision support branch)	Universidade de Aveiro	Aveiro	Public	University	MSc	2020	4	120	No
ICSE	Medical Informatics Engineering	Instituto Politécnico do Cávado e do Ave	Barcelos	Public	Polytechnic	BSc	2018	6	180	No
MNHDPP	Applied Biomedicine (computation branch)	Universidade Católica Portuguesa	Viseu	Private	University	MSc	2021	4	120	No
MNHDPP	Bioinformatics (information technologies branch)	Universidade do Minho	Braga	Public	University	MSc	2011	4	120	No
MNHDPP	Biomedical Sciences	Instituto Universitário de Ciências da Saúde	Paredes	Private	University	BSc	2010	6	180	No
MNHDPP	Healthcare Policies, Management and Evaluation	Universidade do Porto	Porto	Public	University	PGC	2020	2	60	No
MNHDPP	Medical Imaging and Radiotherapy	Instituto Politécnico da Lusofonia	Lisbon	Private	Polytechnic	BSc Hons	2018	8	240	No
MNHDPP	Medical Imaging and Radiotherapy	Instituto Politécnico de Coimbra	Coimbra	Public	Polytechnic	BSc Hons	2014	8	240	No
MNHDPP	Medical Imaging and Radiotherapy	Universidade do Algarve	Faro	Public	Polytechnic	BSc Hons	2014	8	240	No
MNHDPP	Medicine	Universidade do Porto	Porto	Public	University	iMSc	2011	12	360	No
MNHDPP	Medicine (analytical data systems branch)	Universidade do Minho	Braga	Public	University	iMSc	2011	12	360	No

Table 7

**Table:** 

	BMHI (N = 8)	ICSE (N = 7)	MNHDPP (N = 9)	TOTAL (N = 24)
Education System				
Public	8 (100%)	7 (100%)	6 (66.7%)	21 (87.5%)
Private	0 (0%)	0 (0%)	3 (33.3%)	3 (12.5%)
Education Subsystem				
Polytechnic	5 (62.5%)	2 (28.6%)	3 (33.3%)	10 (41.7%)
University	3 (37.5%)	5 (71.4%)	6 (66.7%)	14 (58.3%)
Level				
MSc	5 (62.5%)	4 (57.1%)	2 (22.2%)	11 (45.8%)
PGC	2 (25.0%)	0 (0%)	1 (11.1%)	3 (12.5%)
PhD	1 (12.5%)	0 (0%)	0 (0%)	1 (4.17%)
BSc	0 (0%)	3 (42.9%)	1 (11.1%)	4 (16.7%)
BSc Hons	0 (0%)	0 (0%)	3 (33.3%)	3 (12.5%)
iMSc	0 (0%)	0 (0%)	2 (22.2%)	2 (8.33%)
ECTS Credits				
Mean (SD)	113 (61.6)	146 (32.1)	213 (104)	160 (84.7)
Median [Min, Max]	120 [30.0, 240]	120 [120, 180]	240 [60.0, 360]	120 [30.0, 360]

Table 8

**Table:** 

MAIN AREA	PROGRAMME NAME	INSTITUTION	MANDATORY ECTS				OPTIONAL ECTS			
			BMHI	MEDICAL	INFORMATICS	TOTAL	BMHI	MEDICAL	INFORMATICS	TOTAL
BMHI	Applied Health Sciences - Com. Intervention and Biotech. (biotechnology branch)	Instituto Politécnico de Bragança	7,0	0,0	3,5	81,0	0,0	0,0	0,0	9,0
BMHI	Biomedical Engineering (medical informatics branch)	Universidade do Minho	60,0	0,0	20,0	120,0	0,0	0,0	0,0	0,0
BMHI	Biostatistics and Bioinformatics Applied to Healthcare	Instituto Politécnico do Porto	15,0	0,0	7,5	105,0	0,0	7,5	7,5	15,0
BMHI	Health Data Science	Instituto Politécnico de Lisboa	21,0	4,0	21,0	60,0	0,0	0,0	0,0	0,0
BMHI	Health Data Science (health informatics branch)	Universidade do Porto	33,0	9,0	6,0	240,0	0,0	0,0	0,0	0,0
BMHI	Medical Informatics	Universidade do Porto	81,0	6,0	21,0	108,0	6,0	0,0	6,0	12,0
BMHI	Medical Technology and Healthcare Business	Instituto Politécnico do Porto	15,0	10,0	5,0	120,0	0,0	0,0	0,0	0,0
BMHI	Nursing Information Systems	Escola Superior de Enfermagem do Porto	28,0	0,0	0,0	28,0	6,0	0,0	0,0	2,0
ICSE	Bioengineering in Regenerative and Precision Medicine (precision medicine branch)	Universidade de Lisboa	6,0	6,0	6,0	108,0	0,0	0,0	0,0	12,0
ICSE	Bioinformatics	Instituto Politécnico de Setúbal	5,0	10,0	66,5	180,0	0,0	0,0	0,0	0,0
ICSE	Bioinformatics and Applications to Life Sciences (branch applied computing)	Universidade de Trás-os-Montes e Alto Douro	6,0	6,0	24,0	120,0	0,0	0,0	0,0	0,0
ICSE	Biomedical Engineering	Universidade Nova de Lisboa	6,0	12,0	84,0	171,0	0,0	0,0	0,0	9,0
ICSE	Biomedical Engineering and Biophysics	Universidade de Lisboa	6,0	0,0	12,0	78,0	24,0	6,0	6,0	42,0
ICSE	Clinical Bioinformatics (clinical decision support branch)	Universidade de Aveiro	6,0	12,0	24,0	114,0	0,0	18,0	36,0	6,0
ICSE	Medical Informatics Engineering	Instituto Politécnico do Cávado e do Ave	33,0	15,0	78,0	180,0	0,0	0,0	0,0	0,0
MNHDPP	Applied Biomedicine (computation branch)	Universidade Católica Portuguesa	6,0	60,0	17,0	120,0	0,0	0,0	0,0	0,0
MNHDPP	Bioinformatics (information technologies branch)	Universidade do Minho	5,0	10,0	5,0	120,0	0,0	0,0	0,0	0,0
MNHDPP	Biomedical Sciences	Instituto Universitário de Ciências da Saúde	4,0	56,0	8,0	170,0	0,0	40,0	0,0	10,0
MNHDPP	Healthcare Policies, Management and Evaluation	Universidade do Porto	6,0	18,0	0,0	42,0	9,0	27,0	6,0	18,0
MNHDPP	Medical Imaging and Radiotherapy	Instituto Politécnico da Lusofonia	5,0	28,0	5,0	240,0	0,0	0,0	0,0	0,0
MNHDPP	Medical Imaging and Radiotherapy	Instituto Politécnico de Coimbra	4,0	10,0	3,0	240,0	0,0	0,0	0,0	0,0
MNHDPP	Medical Imaging and Radiotherapy	Universidade do Algarve	4,0	18,0	3,0	240,0	0,0	0,0	0,0	0,0
MNHDPP	Medicine	Universidade do Porto	12,0	330,0	4,0	346,0	10,0	218,0	4,0	14,0
MNHDPP	Medicine (analytical data systems branch)	Universidade do Minho	45,0	300,0	15,0	360,0	0,0	0,0	0,0	0,0

## Changes to the figures

Figures 1, 2, 3 and 4 should be updated as per below (.tiff files attached). Figure titles and captions remain the same.

Figure 1

**Figure:**
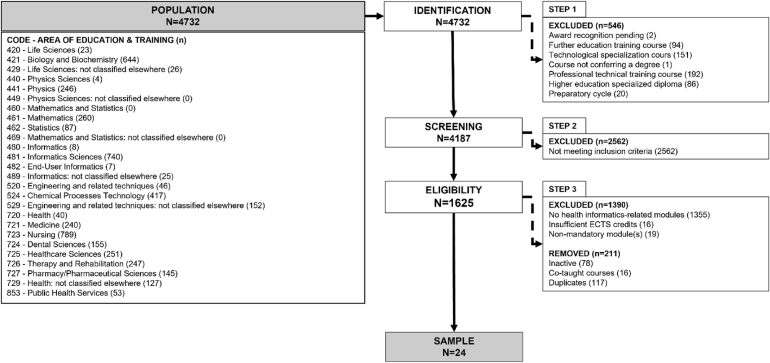

Figure 2

**Figure:**
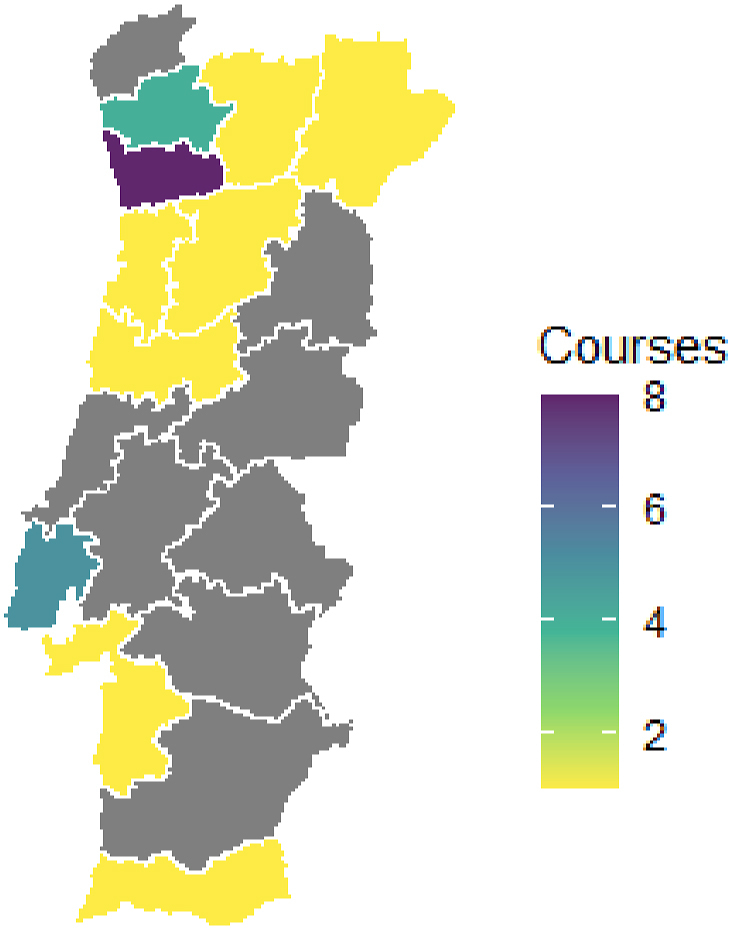

Figure 3

**Figure:**
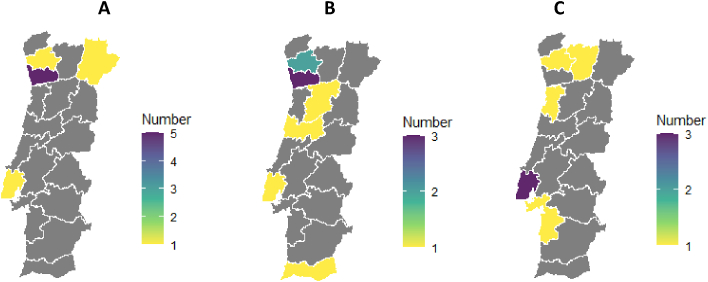

Figure 4

**Figure:**